# Patient Detection of a Drug Dispensing Error by Use of Physician-Provided Drug Samples

**DOI:** 10.1592/phco.22.17.1642.34123

**Published:** 2012-01-17

**Authors:** Elizabeth S Dodds Ashley, Kevin Kirk, Vance G Fowler

**Affiliations:** Division of Infectious Diseases, Duke University Medical CenterDurham, North Carolina; Village Internal MedicineFayetteville, North Carolina

Drug samples commonly are provided by physicians to patients in the outpatient setting. The use of drug samples, however, has come under scrutiny as critics argue that the practice is a marketing tool for the pharmaceutical industry.^1^–^5^ An additional problem facing the medical community is the growing rate of medication errors. Recently, there has been a national call for new methods of identifying and avoiding these frequently disabling events.6 We describe a case in which a medication error was averted through the use of drug samples.

## Case Report

A 63-year-old woman came to her primary care physician with a 2-day history of a swollen right knee. She denied fever, chills, severe pain in her knee, and recent trauma. Medical history was remarkable for degenerative joint disease and paroxysmal atrial fibrillation, for which she took warfarin 7.5 mg/day. Physical examination was remarkable for a cool, nontender, and non-erythematous right knee with a moderate effusion. Arthrocentesis yielded 20–25 ml of straw-colored fluid. Analysis of the joint fluid revealed a white blood cell count of 7.23 × 10^3^/mm^3^ and an erythrocyte count of 320/mm^3^. No crystals were seen by birefringent microscopy, and no organisms were seen with Gram's stain.

The patient was given several sample tablets of rofecoxib 25 mg (Vioxx; Merck & Co., Whitehouse Station, NJ) and was instructed to take one tablet/day for acute exacerbation of degenerative joint disease. She also was given a prescription for Vioxx so that she could continue taking the drug when the samples were consumed. Her symptoms improved with the use of the samples.

Three days later she had her prescription for Vioxx filled at her local pharmacy. Although the pill bottle was labeled as rofecoxib, she noticed that the newly prescribed tablets looked significantly different from the Vioxx samples she had been given. Although the samples were small white pills, the contents of the new prescription were blue diamond-shaped tablets with “VGR 25” inscribed on each side. Concerned because of the difference in appearance between the drug samples and the new tablets, the patient returned the new tablets to the pharmacy, where they were found to be sildenafil citrate 25-mg tablets (Viagra; Pfizer Inc., New York, NY). The drug dispensing error was corrected, and the patient's prescription was replaced.

## Discussion

Medication errors cause as many as 40,000 deaths^7^ and cost nearly 80 billion dollars annually in the United States.^8^ Because of this enormous impact, a recent Institute of Medicine report called for novel ways to reduce rates of medication errors.^6^ This patient's experience resulted in a situation in which a medication error was detected and avoided by the use of drug samples provided by her primary care physician.

The debate over the ethical use of drug samples is likely to continue. Although there is evidence that pharmaceutical companies provide these free drugs in an attempt to influence prescribing patterns of practitioners, often they are used to help treat patients who would not otherwise be able to obtain their drugs. This case report highlights an additional, positive use of drug samples that could help patients visually verify that they receive the intended drug from the pharmacy.
